# The retinoid anticancer signal: mechanisms of target gene regulation

**DOI:** 10.1038/sj.bjc.6602700

**Published:** 2005-07-12

**Authors:** T Liu, A Bohlken, S Kuljaca, M Lee, T Nguyen, S Smith, B Cheung, M D Norris, M Haber, A J Holloway, D D L Bowtell, G M Marshall

**Affiliations:** 1 Children's Cancer Institute Australia for Medical Research, Randwick NSW 2031, Australia; 2 Peter MacCallum Cancer Centre, East Melbourne VIC 8006, Australia; 3 Centre for Children's Cancer and Blood Disorders, Sydney Children's Hospital, High Street, Randwick NSW 2031, Australia

**Keywords:** retinoid, retinoid signalling, gene expression profiling, neuroblastoma

## Abstract

Retinoids induce growth arrest, differentiation, and cell death in many cancer cell types. One factor determining the sensitivity or resistance to the retinoid anticancer signal is the transcriptional response of retinoid-regulated target genes in cancer cells. We used cDNA microarray to identify 31 retinoid-regulated target genes shared by two retinoid-sensitive neuroblastoma cell lines, and then sought to determine the relevance of the target gene responses to the retinoid anticancer signal. The pattern of retinoid responsiveness for six of 13 target genes (RAR*β*2, CYP26A1, CRBP1, RGS16, DUSP6, EGR1) correlated with phenotypic retinoid sensitivity, across a panel of retinoid-sensitive or -resistant lung and breast cancer cell lines. Retinoid treatment of *MYCN* transgenic mice bearing neuroblastoma altered the expression of five of nine target genes examined (RAR*β*2, CYP26A1, CRBP1, DUSP6, PLAT) in neuroblastoma tumour tissue *in vivo*. In retinoid-sensitive neuroblastoma, lung and breast cancer cell lines, direct inhibition of retinoid-induced RAR*β*2 expression blocked induction of only one of eight retinoid target genes (CYP26A1). DNA demethylation, histone acetylation, and exogenous overexpression of RAR*β*2 partially restored retinoid-responsive CYP26A1 expression in RA-resistant MDA-MB-231 breast, but not SK-MES-1 lung, cancer cells. Combined, rather than individual, inhibition of DUSP6 and RGS16 was required to block retinoid-induced growth inhibition in neuroblastoma cells, through phosphorylation of extracellular-signal-regulated kinase. In conclusion, sensitivity to the retinoid anticancer signal is determined in part by the transcriptional response of key retinoid-regulated target genes, such as RAR*β*2, DUSP6, and RGS16.

Retinoids, including retinoic acid (RA), regulate the expression of genes involved in cell proliferation, differentiation, and apoptosis, and are essential for normal embryonic development and health in the adult (Evans and Kaye, 1999). The multiple phenotypic effects of retinoids are mediated, in part, by a classical pathway involving two classes of nuclear receptors: retinoic acid receptors (RARs) and retinoid X receptors (Chambon, 1996). The transcriptional activation, which follows liganded RAR binding to a RA responsive element (RARE), triggers retinoid target gene expression or suppression, and subsequent specific biological effects. In addition to the classical nuclear RA signalling mechanism, covalent binding of RA or RAR to other cellular macromolecules may exert other retinoid effects (Altucci and Gronemeyer, 2001; Balmer and Blomhoff, 2002).

Disruption of normal retinoid signalling has been causally linked to the genesis of several human and experimental cancers (reviewed in (Sun and Lotan, 2002)). Defined mechanisms of retinoid resistance in cancer cells have included increased retinoid catabolism, reduced expression of nuclear retinoid receptors, and repressed transcriptional response of RA target genes (Freemantle *et al*, 2003).

Following retinoid treatment *in vitro*, many cell types upregulate the expression of target genes coding for proteins involved in retinoid binding and metabolism, such as the nuclear RAR*β*2 and retinoic acid hydroxylase (CYP26A1) (Sonneveld *et al*, 1998; Cheung *et al*, 2003). The RA-responsive transcription of RAR*β*2 is frequently lost in breast, lung, prostate, cervical, and oral carcinoma (Sun and Lotan, 2002; Freemantle *et al*, 2003). We have previously shown that derepression, or exogenous overexpression, of RAR*β*2 can restore retinoid responsiveness of some cells (Cheung *et al*, 2003). We, and others, have also provided evidence that unliganded RAR*β*2 may have an additional role as a tumour suppressor gene (Houle *et al*, 1993; Cheung *et al*, 1998). However, target genes of liganded or unliganded RAR*β*2 have not yet been defined. CYP26A1, on the other hand, leads to RA catabolism.

Here, we have defined a group of 31 RA-regulated genes in RA-responsive neuroblastoma cells *in vitro*. A subset of these target genes was also regulated *in vivo*, and correlated with phenotypic retinoid sensitivity in lung and breast cancer cells *in vitro*. RA-induced expression of liganded RAR*β*2 directly regulated CYP26A1 in RA-sensitive and -resistant neuroblastoma, lung and breast cancer cells. Promoter methylation and histone deacetylation, in part, explained the lack of retinoid responsiveness of target genes in some RA-resistant cells. Synchronous induction by RA of two target genes, known to be mitogen-activated protein kinase (MAPK) extracellular-signal-regulated kinase (ERK) signaling pathway inhibitors, was required to mediate retinoid effects on cell proliferation, through reduction of ERK phosphorylation.

## MATERIALS AND METHODS

### Cell culture

Human neuroblastoma BE(2)-C and SH-SY5Y cell lines were generously supplied by Dr J Biedler (Memorial Sloan-Kettering Cancer Center, NY, USA). Human lung Calu-6 and SK-MES-1, and mammary T47D and MDA-MB-231 epithelial cancer cells were obtained from the American Type Culture Collection (Manassas, VA, USA). All cells were cultured in Dulbecco's modified Eagle's medium supplemented with L-glutamine and 10% fetal calf serum. (aRA) all-trans RA, 13-*cis*-RA, trichostatin A (TSA, an inhibitor of histone deacetylase) (Sigma, St Louis, MO, USA) were solubilised in ethanol. The DNA demethylation agent aza-CdR (5-aza-2-deoxycytidine) (Sigma) was dissolved in water.

### cDNA microarray

After treatment with control or 10 *μ*M aRA for 1, 24 h, 3 or 7 days, BE(2)-C and SH-SY5Y cells were lysed, and RNA extracted with the standard guanidinium/phenol/chloroform method. Direct labelling cDNA microarray experiments were carried out as described previously (Boussioutas *et al*, 2003). Results from three independent microarray hybridisations, with probes synthesised using RNA from three independent cell treatment experiments, were analysed. An arbitrary postnormalisation cutoff of two-fold up- or downregulation was used to define significant differential gene expression.

### Semiquantitative competitive reverse transcription–polymerase chain reaction (RT–PCR)

Confirmation of microarray data in the two neuroblastoma cell lines, in mammary and lung cancer cell lines, and in neuroblastoma tissues was carried out with competitive RT–PCR with RNAs from three independent cell culture experiments and from 12 mice. All results of RT–PCR for each gene were analysed from at least three PCR results. The competitive PCR techniques have been previously described (Norris *et al*, 1996; Cheung *et al*, 2003), which involved determining a ratio between the level of expression of a target gene and that of the house-keeping gene *β*2-microglobulin (*β*2M) in total RNA samples. Fold induction of a target gene by RA was calculated by ascribing the ratio as 1.0 for control-treated samples. Specific primers are listed in Table 1.

### Small interfering RNA (siRNA) designing

Small interfering RNA for RAR*β*2 was purchased from Dharmacon (Dharmacon Research, Lafayette, CO, USA) (Catalogue number: M003438-00-05). The sequences of the siRNA were patented and cannot be published according to Dharmacon. Small interfering RNA target sequences of 21 nucleotides for DUSP6 and RGS16 were identified using the principles described by Elbashir *et al* (2001). Three suitable targets were found for DUSP6: AAGAACTGTGGTGTCTTGGTA, AAGCTCAATCTGTCGATGAAC, and AAGTGCGGAATTGGTTAATAC; and three targets for RGS16: AAGATCCGATCAGCTACCAAG, AAACTTCTCAGAAGATGTGCT, and AACAAGGCAGAAAAGGATCCT. Double-stranded siRNA oligos were *in vitro* transcribed with Ambion Silencer siRNA Construction Kit (Ambion, Austin, TX, USA) according to the manufacturers’ instructions. Scrambled siRNAs with the same GCAT content as target siRNAs, but different sequences, were also *in vitro* transcribed, and it was confirmed that all siRNAs did not resemble any other mRNA (<15/21).

### Transient transfection

#### Plasmid cDNA

RAR*β*2 and control plasmids were kindly provided by Professor P Chambon (INSERM, Strasbourg, France). Transient transfection was performed using Superfect transfection reagent (Qiagen, Clifton Hill, Victoria, Australia) in MDA-MB-231 and SK-MES-1 cells, or Lipofectamine 2000 reagent (Invitrogen, Carlsbad, CA, USA) in SH-SY5Y cells. After 8 h, cells were treated with 10 *μ*M aRA or control. Transfection efficiency and its effect on potential target gene expression were assessed by RT–PCR with RNA extracted 24 h or 3 days after aRA treatment.

#### Small interfering RNA

SH-SY5Y, Calu-6, and T47D cells were transfected with 100 nM siRNA using Lipofectamine 2000 reagent according to Invitrogen. After 8 h, cells were treated with 10 *μ*M aRA or control. RNA and/or protein were extracted 48 h later. Transfection efficiency and its effect on potential target gene expression was assessed by RT–PCR and/or immunoblot.

### Immunoblot analysis

Cells were lysed, and protein extracted and analysed by sodium dodecyl sulphate–polyacrylamide gel electrophoresis. For DUSP6 and RGS16 studies, membranes were probed with either rabbit anti-RGS16 antibody (diluted 1 : 500) or goat anti-DUSP6 antibody (1 : 500) (Santa Cruz Biotechnology, CA, USA) followed by horseradish peroxidase (HRP)-conjugated anti-rabbit (1 : 2000) or anti-goat antiserum (1 : 2000) (Pierce, Rockford, IL, USA), respectively. The membranes were then incubated with fluorescence-conjugated ECL Plus (Amersham) and scanned with a Typhoon Scanner (Amersham). Membranes were lastly reprobed with anti-*β*-actin antibody (Pierce) as a loading control. Comparative protein expression was semiquantified by analysing target protein bands *vs β*-actin with ImageQuant software (Amersham).

For the MAPK ERK phosphorylation study, membranes were probed with mouse antiphosphorylated ERK1/2 antiserum (1 : 2000) (Upstate Biotech, MA, USA), followed by incubation with HRP-conjugated anti-mouse antiserum (1 : 2000) (Pierce). Equal loading of protein was confirmed by probing the membranes for total ERK protein with rabbit anti-ERK1/2 (nonphosphorylated) antiserum (1 : 2000) (Upstate Biotech), followed by incubation with HRP-conjugated anti-rabbit antiserum (1 : 2000) (Pierce).

### Cell proliferation assay

The cell proliferation assay was carried out with the *In Situ* Cell Proliferation Kit, FLUOS (Roche Applied Science, Switzland), according to the manufacturer's instructions. SH-SY5Y cells transfected with scrambled siRNA, DUSP6 siRNA, RGS16 siRNA, a combination of DUSP6 and RGS16 siRNAs, or a combination of scrambled siRNAs, were treated with 10 *μ*M aRA for 64 h, followed by incubation with 10 *μ*M 5-bromo-2′-deoxyuridine (BrdU) for 45 min. After fixation in 70% ethanol overnight and denaturation with hydrochloric acid, cells were incubated with an anti-BrdU-FLUOS antibody and analysed on a flow cytometer (FACScan, Becton Dickinson, NJ, USA). Cells incubated with solvent instead of BrdU, but incubated with anti-BrdU-FLUOS were used as control for autofluorescence and background staining. Low and high fluorescence regions were defined for quantitation: low fluorescent regions comprised >95% of control cells (BrdU-negative or nonproliferating cells), whereas high fluorescent regions contained proliferating BrdU-positive cells. The percentage of BrdU-negative and -positive cells over total cell population was compared among target gene siRNA-, and scrambled siRNA-transfected cells treated with 10 *μ*M aRA.

### Animal model studies

The generation of the *MYCN* transgenic mice has been described previously (Weiss *et al*, 1997). All homozygote *MYCN* transgenic mice developed neuroblastoma at the age of 6–7 weeks (Weiss *et al*, 1997). Once a tumour was palpable in the abdomen, homozygote *MYCN* transgenic mice were randomised and treated with either solvent control (*n*=6) or 13-*cis*-RA at the dosages of 0.72–1.43 mmol 24 h^−1^ (*n*=6) via an Alzet micro-osmotic pump (ALZET, Cupertino, CA, USA). The pump was surgically implanted subcutaneously at the back, which caused minimal discomfort to the mice (Burkhart *et al*, 2003). The drugs were delivered at a constant rate of 0.5 *μ*L h^−1^ (Burkhart *et al*, 2003). Mice were monitored once every day from the commencement of treatment and killed 5 days after treatment. Tumour tissues were removed and RNA was extracted. Competitive RT–PCR was carried out to examine whether RA target genes identified *in vitro* were modulated by RA *in vivo* as well. All animal experimental procedures were approved by the University of New South Wales Animal Care and Ethics Committee, and were consistent with United Kingdom Coordinating Committee on Cancer Research guidelines for the welfare of animals in experimental neoplasia. Compared with untreated animals and animals treated with control, 13-cis-RA treatment did not induce any significant side effects.

### Statistical analysis

All data for statistical analysis were calculated as mean±standard error. Differences were analysed for significance using ANOVA among groups. A probability value of 0.05 or less was considered significant.

## RESULTS

### Microarray data and validation of a subset of differentially expressed RA target genes

To identify RA-regulated target genes in neuroblastoma cells, we performed triplicate microarray experiments comparing gene expression in BE(2)-C and SH-SY5Y neuroblastoma cell lines treated continuously with 10 *μ*M aRA for 1, 24 h, 3 or 7 days. SH-SY5Y cell line is nonamplified, and the BE(2)-C cell line is amplified, for the oncogene *MYCN*, an important determinant of the RA response *in vitro* and patient prognosis *in vivo* (Bordow *et al*, 1998; Nguyen *et al*, 2003).

**Figure 1:**
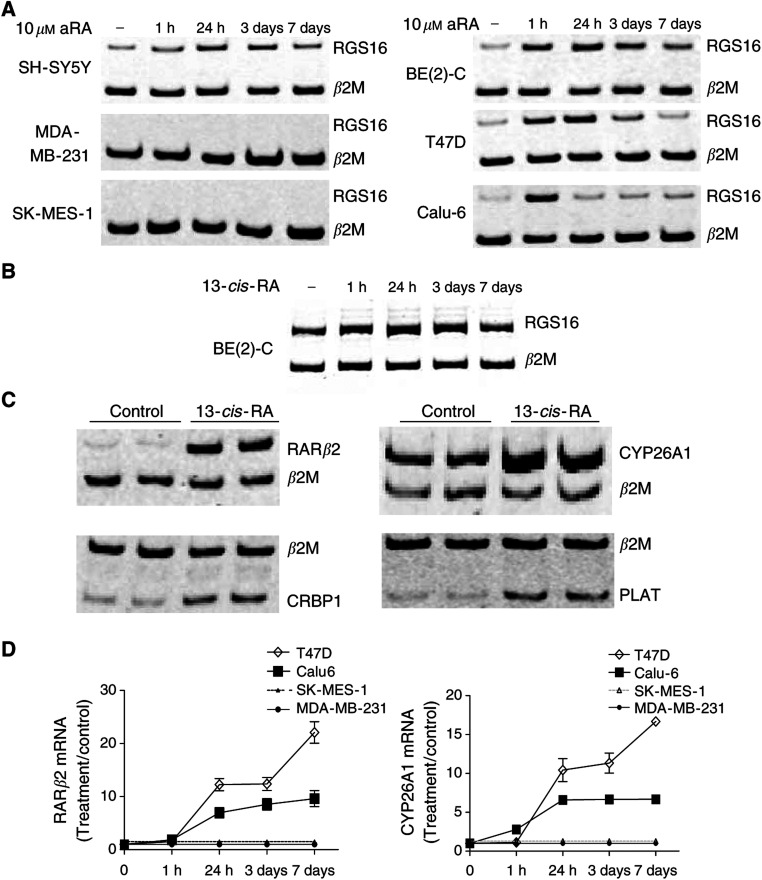
Induction of target gene expression by RA in neuroblastoma (SH-SY5Y, BE(2)-C), lung (SK-MES-1, Calu-6) and breast (MDA-MB-231, T47D) cancer cell lines, and neuroblastoma tissues. cDNA samples from cultured cells treated with 10 *μ*M aRA (**A** and **D**), or 13-*cis*-RA (**B**) or solvent control at various time points, and duplicate cDNA samples from neuroblastoma arising in *MYCN* transgenic mice treated with 13-*cis*-RA or control (**C**) were subjected to independent competitive RT–PCR analyses using trans-intron PCR primers, together with housekeeping gene *β*2M primers. An equal aliquot of PCR product was then electrophoretically size-fractionated on a polyacrylamide gel as shown (**A**, **B** and **C**). Fold induction of a target gene by RA in RA-treated samples was calculated by ascribing the ratio between the level of expression of a target gene and that of *β*2M as 1.0 for control-treated samples (**D**).

Microarray slides with 4500 cDNA clones, representing predominantly genes with known functions, were hybridised with cDNA from aRA- or control-treated cells. In total, 31 genes were up- or downregulated by ⩾2-fold by aRA in both cell lines, at one or more time points in triplicate (*P*<0.05) (listed in Table 2 according to the time course of changes in gene modification). This list of RA-regulated targets included genes coding for proteins known to be involved in: (i) retinoid binding and metabolism (RAR*β*2, cellular retinoid-binding protein 1 (CRBP1), and CYP26A1); (ii) the MAPK signalling pathway (regulator of G-protein signalling 16 (RGS16) and dual specificity phosphatase 6 (DUSP6)); (iii) cell structure and differentiation (filamin B (FLNB), c-*RET* proto-oncogene (RET), *α* polypeptide Cu^2+^ transporting ATPase (ATP7A), and *δ*-like 1 homolog (Drosophila) (DLK1)); and (iv) angiogenesis or cancer invasion (early growth response protein 1 (EGR1) and tissue plasminogen activator (PLAT)). Among the 31 genes, a classical RARE could be found in the promoter region of RAR*β*2, CYP26A1, CRBP1 and PLAT (Chambon, 1996; Sun and Lotan, 2002). While RGS16 expression was upregulated at 1 h after aRA (Table 2, Figure 1A), all other RA target genes showed a more gradual change in expression at 1, 3, and 7 days after commencement of aRA. Only four of 31 target genes demonstrated downregulated expression patterns after aRA treatment. In addition to those genes listed in Table 2, a further 21 genes exhibited ⩾2-fold change in expression in only one of the two neuroblastoma cell lines (data not shown). To validate the microarray data, semiquantitative, competitive RT–PCR was employed to assess expression of 11 selected genes shared by the two cell lines (Table 2). The results of RT–PCR were consistent with the microarray data in 10 out of 11 genes, with TIA1 as the only exception. Examples of the RT–PCR results were shown in Figure 1A.

We next examined whether target gene responses were specific for aRA, compared with other isomeric retinoid compounds, such as 13-*cis*-RA, which is in current clinical use in neuroblastoma patients (Matthay *et al*, 1999). Competitive RT–PCR analysis confirmed that all eight target genes tested were upregulated by 13-*cis*-RA in a manner similar to aRA in BE(2)-C cells (Figure 1B). More specifically, RAR*β*2, CYP26A1, CRBP1, RGS16, DUSP6, FLNB, PLAT, and RET were upregulated by 5.0-, 107.1-, 1.7-, 1.8-, 1.9-, 4.5-, 2.6-, and 1.6-fold, respectively, 3 days after 10 *μ*M 13-*cis*-RA treatment (*P*<0.05 in all cases).

### Regulation of RA target genes in neuroblastoma tumour tissue *in vivo*

To assess whether the RA target genes identified *in vitro* were also modulated by RA *in vivo*, we treated homozygous *MYCN* transgenic mice with palpable abdominal neuroblastoma with 13-*cis*-RA (0.72–1.43 mmol 24 h^−1^) or solvent control, and compared target gene expression by RT–PCR (Figure 1C). While no significant side effects were observed, the 5-day therapy with 13-*cis*-RA did not reduce tumour size, when compared with solvent-treated mice. Competitive RT–PCR showed that 13-*cis*-RA upregulated the expression of RAR*β*2 by 7.6±0.8-fold, which was comparable to the *in vitro* data. In contrast, CYP26A1 was induced by only 2.0±0.3-fold (*P*<0.05), compared to induction by more than 100-fold in 13-*cis*-RA-treated BE(2)-C cells *in vitro*. Other RA target genes which were also upregulated *in vivo* included CRBP1 by 1.8±0.1-fold, DUSP6 by 2.5±0.5-fold, and PLAT by 1.9±0.2-fold (*P*<0.05). However, 13-*cis*-RA did not significantly modulate RET, RGS16, FLNB, and EGR1 expression in the tissue samples from tumour-bearing animals treated for 5 days.

### Retinoid responsiveness of specific RA target genes correlates with the phenotypic retinoid response in neuroblastoma, lung and breast cancer cells

We next asked whether the patterns of change in RA target gene expression seen in neuroblastoma cells were shared by other cancer types. RA-sensitive lung (Calu6) and breast (T47D) cancer cell lines, and RA-resistant lung (SK-MES-1) and breast (MDA-MB-231) cancer cells were treated with control or 10 *μ*M aRA for 1, 24 h, 3 or 7 days. Competitive RT–PCR was carried out to assess changes in the expression of the 13 target genes with cDNA from three independent cell culture and treatment experiments. As shown in Table 3 and Figure 1A and D, four of 12 target genes (RAR*β*2, CYP26A1, CRBP1, and RGS16) were upregulated by more than two-fold in both the RA-sensitive lung and breast cancer cells. However, RGS16, RAR*β*2, and CYP26A1 were not detectable and not RA inducible in both of the RA-resistant breast and lung cancer cells. DUSP6 was induced in T47D cells only, while EGR1 was upregulated in Calu6 cells only. Surprisingly, RET and PLAT were downregulated by 10- and 2.2-fold in RA-sensitive Calu-6 lung cancer cells, whereas both target genes were upregulated in both neuroblastoma cell lines. In contrast, ATP7A, DLK1, CRBP1, and SMAD3 were not modulated by aRA in RA-sensitive lung and breast cancer cells.

### DNA demethylation and histone acetylation partially restore RAR*β*2 and CYP26A1 gene expression in RA-resistant breast, but not lung cancer cells

**Figure 2:**
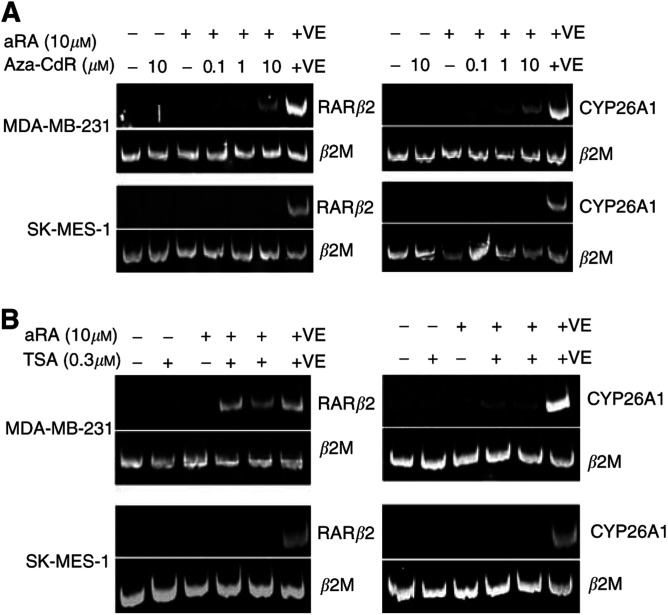
The effect of demethylation and acetylation on aRA-induced RAR*β*2 and CYP26A1 expression in RA-resistant cancer cells. RAR*β*2 and CYP26A1 expression was determined by noncompetitive RT–PCR, with expression of housekeeping gene *β*2M as an internal control. (**A**) MDA-MB-231 and SK-MES-1 cells were treated with control, aRA, and/or various concentration of aza-CdR for 3 days. (**B**) The cells were treated with control, aRA, and/or 0.3 *μ*M TSA for 3 days. The last lane of each gel (+VE) contained a positive control from RA-sensitive Calu-6 cells treated with 10 *μ*M aRA for 3 days.

We observed a close correlation between the expression pattern of RAR*β*2 and CYP26A1 in response to retinoid (Tables 2 and 3). This led us to hypothesise that these two target genes may have a common mechanism of transcriptional repression in RA-resistant cells. To determine whether DNA methylation of regulatory elements resulted in the repression of RAR*β*2 and CYP26A1, RA-resistant lung and breast cancer cells were treated with the demethylating agent, aza-CdR. Treatment of cells with 0.1, 1.0, and 10 *μ*M aza-CdR, in combination with 10 *μ*M aRA for 3 days, did not restore RAR*β*2 and CYP26A1 expression in the aRA-resistant cells when analysed by competitive RT–PCR. Minimally expressed genes may be out-competed by the *β*2M gene in a competitive RT–PCR (Freeman *et al*, 1999). Therefore, RAR*β*2 and CYP26A1 expression was further analysed by noncompetitive RT–PCR. In noncompetitive RT–PCR, we detected weak RAR*β*2 and CYP26A1 (Figure 2A) expression in the RA-resistant breast cancer cell line, MDA-MB-231, after treatment with aza-CdR and aRA, in a dose-dependent manner. However, both RAR*β*2 and CYP26A1 (Figure 2A) expression levels in treated MDA-MB-231 cells were relatively low compared to the levels seen in RA-sensitive lung cancer-positive control cells, Calu-6, also treated with 10 *μ*M aRA for 3 days. CYP26A1 and RAR*β*2 transcripts were not detected by either competitive or noncompetitive RT–PCR in the RA-resistant lung cancer cell line. These data indicate that DNA methylation is one factor contributing to the repression of RAR*β*2 and CYP26A1 transcription in MDA-MB-231 cells. However, demethylation was insufficient to derepress transcription to a level equivalent to that seen in a RA-sensitive cell line, for both RAR*β*2 and CYP26A1.

Since the level of histone acetylation, and conversely deacetylation, can influence gene transcription, the effect of the histone deacetylase (HDAC) inhibitor, TSA on RAR*β*2 and CYP26A1 expression in RA-resistant lung and breast cancer cells was investigated. Cells were treated with 0.3 *μ*M TSA in combination with aRA (Sirchia *et al*, 2002) for 24 h and 3 days, and analysed for RAR*β*2 and CYP26A1 (Figure 2B) expression. CYP26A1 and RAR*β*2 transcripts were detected in MDA-MB-231 cells after treatment with TSA and aRA, yet at a much lower level than in RA-treated Calu-6-positive control cells. CYP26A1 and RAR*β*2 transcripts were not detected in SK-MES-1 cells. These observations together with the data from the demethylation experiments suggest that SK-MES-1 and MDA-MB-231 cells have different mechanisms of transcriptional repression for RAR*β*2 and CYP26A1, despite having a similar pattern of resistance to RA-induced transcription.

### Liganded RAR*β*2 regulates RA-induced CYP26A1 expression

**Figure 3:**
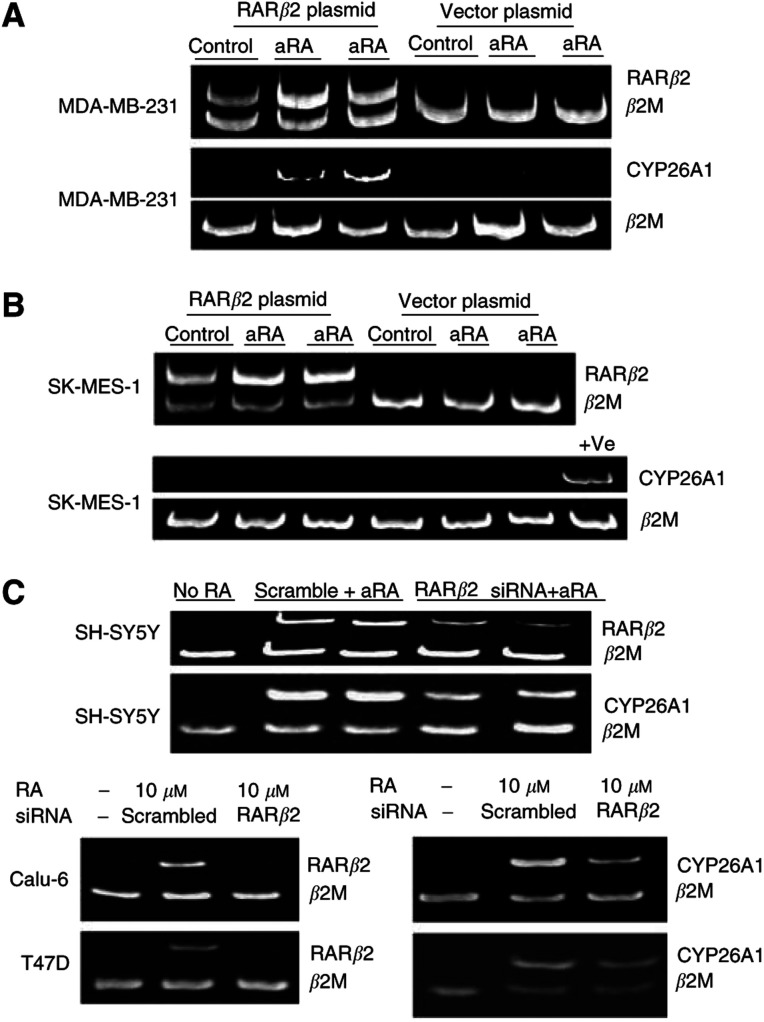
Liganded RAR*β*2 modulates CYP26A1 expression. RAR*β*2 gene expression was analysed with the standard competitive RT–PCR, and CYP26A1 expression was analysed by competitive RT–PCR in RA-sensitive SH-SY5Y, Calu-6, and T47D cells and noncompetitive RT–PCR in RA-resistant cells, with housekeeping gene *β*2M as an internal control. (**A**, **B**) CYP26A1 transcription was determined in MDA-MB-231 (**A**) or SK-MES-1 cells (**B**) transiently transfected with RAR*β*2 cDNA plasmid or vector plasmid and treated with control or 10 *μ*M aRA for 24 h (Lanes 2 and 5) or 3 days (lanes 3 and 6). Last lane in (**B**) contained the positive control from Calu-6 cells treated with 10 *μ*M aRA for 3 days. (**C**) RAR*β*2 and CYP26A1 expression was analysed in SH-SY5Y, Calu-6, and T-47D cells transfected with scramble siRNA or RAR*β*2 siRNA and treated with 10 *μ*M aRA for 48 h.

We next examined whether restoring RAR*β*2 expression could restore CYP26A1 transcription in RA-resistant cells. MDA-MB-231 and SK-MES-1 cells were transiently transfected with a RAR*β*2 cDNA expression vector or empty vector control, and treated with 10 *μ*M aRA for 24 h or 3 days following transfection (Figure 3A and B). Results from a noncompetitive RT–PCR demonstrated a two-fold increase in CYP26A1 levels in the RAR*β*2-transfected MDA-MB-231 cells, compared to cells transfected with the empty vector (Figure 3A). However, the SK-MES-1 cells transfected with RAR*β*2 did not demonstrate retinoid-inducible CYP26A1 expression (Figure 3B).

To further examine the possibility that RAR*β*2 directly regulated CYP26A1 transcription in RA-sensitive cells, SH-SY5Y cells were transiently transfected with an RAR*β*2 cDNA expression vector or empty vector. Reverse transcription–polymerase chain reaction showed that RAR*β*2 plasmid transfection upregulated RAR*β*2 gene expression by about eight-fold, yet CYP26A1 expression was still undetectable, without RA treatment. In a separate experiment, SH-SY5Y cells, Calu-6 lung and T47D breast cancer cells were transiently transfected with scrambled siRNA or specific RAR*β*2 siRNA and then treated with 10 *μ*M aRA for 48 h. The RAR*β*2 siRNA reduced RA-induced RAR*β*2 transcription by about 80% in three independent transfection experiments (Figure 3C). Retinoic acid-induced CYP26A1 expression was downregulated by RAR*β*2 siRNA by 36.47±6.57% in SH-SY-5Y cells, 39.85±5.38% in Calu-6 cells, and 24±6.5% in T47D cells (*P*<0.001). Since both RAR*β*2 and CYP26A1 gene expression was not detectable without aRA treatment in all aRA-sensitive and -resistant cancer cells tested, we did not carry out RAR*β*2 siRNA transfection experiments without treatment with aRA. These results indicated that RA-induced CYP26A1 expression required retinoid liganded RAR*β*2, in RA-sensitive and some RA-resistant cells. Interestingly, transient transfection of RAR*β*2 siRNA did not repress other RA-inducible gene expression (DUSP6, RGS16, FLNB, CRBP1, RET, PLAT).

### Synchronous RA-induced expression of DUSP6 and RGS16 is required for the antiproliferative effect of RA

**Figure 4:**
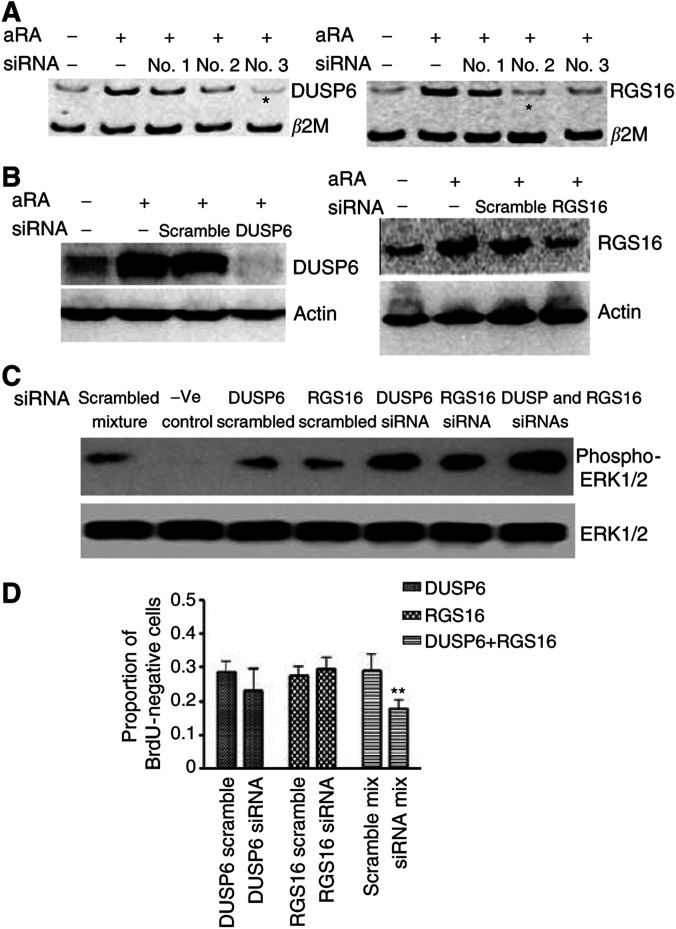
Synchronous expression of both DUSP6 and RGS16 contributed to RA-induced growth inhibition. (**A**) DUSP6 and RGS16 gene expression was analysed with competitive RT–PCR with the housekeeping gene *β*2M as an internal control with samples from SH-SY5Y cells transfected with DUSP6 or RGS16 siRNA or scrambled siRNA and treated with 10 *μ*M aRA for 48 h. ^*^Indicates the siRNAs of choice for protein and functional studies. (**B**) DUSP6 and RGS16 protein was analysed by Western blot with samples from SH-SY5Y cells transfected with scrambled siRNA, DUSP6, or RGS16 siRNA and treated with 10 *μ*M aRA or control solvent for 48 h. *β*-Actin protein was used as a loading control. (**C**) Phosphorylated ERK1/2 was analysed by Western blot with samples from SH-SY5Y cells transfected with scrambled, DUSP6, RGS16 siRNA, or siRNA combinations and treated with 10 *μ*M aRA for 60 h. Total ERK1/2 protein was used as a loading control. (**D**) BrdU incorporation into proliferating cells was analysed in SH-SY5Y cells after transfection with scrambled or target gene siRNAs plus treatment with 10 *μ*M aRA for 64 h. BrdU-positive cells treated with vehicle solvent and transfected with scrambled siRNA were artificially set as 100%. Error bar represented standard error. ^**^Indicated statistical significant difference (*P*<0.05).

DUSP6 and RGS16 are both inhibitors involved in the ERK MAPK signalling pathway, RGS16 at the level of Ras G proteins (Buckbinder *et al*, 1997; Chen *et al*, 2001) and DUSP6 at the level of ERK proteins (Groom *et al*, 1996; Sah *et al*, 2002; Nakagawa *et al*, 2003). Since the growth inhibitory effect of RA is at least partly due to reduction in ERK phosphorylation (Nakagawa *et al*, 2003), we examined the hypothesis that RA-induced proliferative arrest was dependent on increased expression of DUSP6 or RGS16. Three DUSP6 and three RGS16 siRNA duplexes were *in vitro* transcribed, and transfected into SH-SY5Y cells, followed by 10 *μ*M aRA treatment. Competitive RT–PCR showed that the DUSP6 siRNA targeting AAGTGCGGAATTGGTTAATAC, and the RGS16 siRNA targeting AACAAGGCAGAAAAGGATCCT, were the most efficient siRNAs at reducing the expression of each gene (Figure 4A). These siRNAs were, therefore, chosen in all further experiments for protein and cell proliferation studies. As shown in Figure 4B, 48 h of treatment with 10 *μ*m aRA induced RGS16 protein by two-fold, and DUSP6 protein more dramatically from only just detectable, compared with solvent control. At the same time point, RGS16 siRNAs effectively counteracted RA-responsive RGS16 overexpression, while DUSP6 siRNA abolished RA-responsive DUSP6 induction.

To determine the roles of DUSP6 and RGS16 in MAPK ERK dephosphorylation, cell lysates from SH-SY5Y cells transfected with scrambled, DUSP6, and/or RGS16 siRNAs with or without 10 *μ*M aRA treatment for 60 h were subjected to ERK and phosphorylated ERK immunoblot. Without aRA intervention, DUSP6 and/or RGS16 siRNA transfection did not have an effect on ERK phosphorylation. Compared with scrambled siRNA counterparts, DUSP6 siRNA increased ERK phosphorylation by about 2.5-fold, while RGS16 siRNA induced ERK phosphorylation by 1.4-fold (Figure 4C). When cells were cotransfected with siRNAs against DUSP6 and RGS16, we observed an additive effect on ERK phosphorylation of a further 1.6-fold compared with DUSP siRNA alone, or four-fold compared with scrambled siRNA mixture (Figure 4C). All siRNAs did not show any effect on total ERK protein expression.

In the cell proliferation studies, BrdU incorporation by SH-SY5Y cells transfected with scrambled siRNA was decreased after 60 h of aRA treatment, compared with solvent control. We did not observe a significant effect of transfection of DUSP6 siRNA or RGS16 siRNA alone, compared with scrambled siRNA, on BrdU uptake (Figure 4D). In contrast, transfection of a combination of DUSP6 and RGS16 siRNAs reduced the proportion of BrdU-negative cells due to aRA treatment, and reduced the effect of aRA on BrdU incorporation by more than 40% (*P*<0.05). These findings indicated that combined inhibition of the MAPK at two levels mediated the retinoid effects on cell proliferation. As DUSP6 protein was hardly detectable without RA treatment, the effect of siRNA in the cell proliferation assay was not carried out without RA treatment.

## DISCUSSION

In this study, we used cDNA microarray to identify 31 RA-regulated target genes shared by two RA-sensitive neuroblastoma cells lines, and then evaluated the relevance of RA-induced changes in target gene expression to the RA anticancer effect. Our data suggest that RA-induced changes in the expression of RAR*β*2, CYP26A1, CRBP1, RGS16, DUSP6, and EGR1 may be necessary for the retinoid anticancer signal in neuroblastoma, breast and/or lung cancer cells. However, we found little evidence for a direct signalling relationship among these genes, except that RA-induced CYP26A1 expression is partly modulated by RAR*β*2. Our data showed clearly that exogenous overexpression of RAR*β*2 did not induce CYP26A1 expression in the absence of RA ligand, even in RA-sensitive cancer cells. However, forced overexpression of RAR*β*2 activated CYP26A1 transcription in RA-resistant breast cancer cells when the RAR*β*2 ligand, RA, was added. Consistently, knocking down of RA-induced RAR*β*2 expression partly blocked RA-induced CYP26A1 expression across RA-sensitive neuroblastoma, lung and breast cancer cells. This inter-relationship is in agreement with earlier studies, which showed that exogenous RAR*β*2 overexpression in RA-resistant HCT-116 colon cancer cells partially restored RA-induced CYP26A1 expression (Sonneveld *et al*, 1998). Our data indicate that the close link between RAR*β*2 and CYP26A1 expression patterns may be explained by the dependence of CYP26A1 expression on liganded RAR*β*2. Additionally, we found that 13-*cis*-RA only marginally upregulated CYP26A1 expression in neuroblastoma tissues *in vivo*, while inducing RAR*β*2 expression to a similar extent as *in vitro*. This observation suggests that there may be other mechanisms limiting the extent to which 13-*cis*-RA liganded RAR*β*2 can induce CYP26A1 *in vivo*.

DNA methylation and histone deacetylation of the RAR*β*2 gene promoter region have been proposed to result in RAR*β*2 silencing and RA resistance (Sirchia *et al*, 2002; Suh *et al*, 2002). Our study confirmed these findings and found, for the first time, that both DNA methylation and HDAC contribute to CYP26A1 silencing in MDA-MB-231 cells. In contrast, neither DNA demethylation nor the HDAC inhibitor had any effect on RA-induced CYP26A1 or RAR*β*2 expression in SK-MES-1 cells. The RAR*β*2 gene promoter has been shown to be hypermethylated and hypoacetylated in SK-MES-1 cells, and to be resistant to DNA demethylation and histone acetylation (Suh *et al*, 2002). The CYP26A1 gene promoter may also have been resistant to demethylating or acetylating agents due to as yet undefined mechanisms.

This study, for the first time, identified DUSP6 and RGS16 as novel RA target genes, and found that synchronous knock down of RA-induced DUSP6 and RGS16 expression synergistically/additively increased MAPK ERK phosphorylation and partly blocked RA-induced growth inhibition, although decreased expression of either protein alone was insufficient to effect cell proliferation. DUSP6 encodes a dual-specificity phosphatase specific for ERK (Groom *et al*, 1996), a key effector MAPK involved in the RAS-GTP signal transduction pathway (Hunter, 1995; Becker, 2004). DUSP6 dephosphorylates activated ERK and blocks the growth-stimulatory signals (Furukawa *et al*, 2003; Furukawa and Horii, 2004). RGS16, on the other hand, acts as a mechanism for p53 to exert cellular growth control and acts as a negative feedback regulator in response to mitogenic signals (Buckbinder *et al*, 1997). RGS16 inhibits ERK activation upstream of the RAS-RAF-MEK-ERK pathway by enhancing GTPase-activating protein function and inactivating RAS-GTP (Buckbinder *et al*, 1997; Chen *et al*, 2001). Various inhibitors of RAS-RAF-MEK-ERK signalling pathway have been proven to inhibit cancer cell growth and survival, and are already in phase II clinical trials for the treatment of various cancers (Sebolt-Leopold and Herrera, 2004). DUSP6 and RGS16 transcriptional upregulation inhibits tumour cell proliferation and ERK phosphorylation, and serves as a negative regulatory mechanism to prevent further ERK phosphorylation. Our data therefore indicates that RA-induced upregulation of DUSP6 and RGS16 inhibits tumour cell proliferation, through acting on two levels of RAS-RAF-MEK-ERK signalling pathway and eventually synergistically reducing ERK phosphorylation.

In conclusion, we have demonstrated both classical and novel mechanisms by which RA-regulated target gene expression patterns may relate to the retinoid anticancer signal. Our findings have confirmed the importance of previously recognised signalling molecules such as RAR*β*2, and have identified novel roles for MAPK signal inhibitory proteins DUSP6 and RGS16 in mediating the retinoid anticancer effect.

## Figures and Tables

**Table 1:** Primers for semiquantitative competitive RT–PCR for human (Hs) cell lines and mouse (Mm) tissues

**Gene**	**Forward primer**	**Reverse primer**
*β*2M (Hs)	ACCCCCACTGAAAAAGATGA	ATCTTCAAACCTCCATGATG
RAR*β*2 (Hs)	CTACACTGCGAGTCCGTCTT	CAGAGCTGGTGCTCTGTGTT
CYP26A1 (Hs)	CAGCCACATCTCTGATCACT	AAGTTGTTCCAAAATTTCCA
CRBP1 (Hs)	AGGCATAGATGACCGCAAGT	TCATCTCTAGGTGCAGCTCA
CRABP1 (Hs)	GATCCACTGCACCCAAACTC	AAGCCAGCTGCCTTCATTCC
RGS16 (Hs)	GTGGGGCAGTAAACACAGCA	GAACTCCAGGTTCTCCTCAC
DUSP6 (Hs)	GTTTTTCCCTGAGGCCATTT	TAGGCATCGTTCATCGACAG
TIA1 (Hs)	AAGGATTTGGAGTAGATCAA	AGTCCCGGCTCACTGTGTTT
RET (Hs)	GGAAAAGTGGTCAAGGCAAC	ATGTGGGTGGTTGACCTGCT
FLNB (Hs)	AGAGCATCACCCGCACCAGT	GCACAATCTCTGCCTCAGTC
EGR1 (Hs)	CAGCAGCAGCAGCACCTTCA	CGATGTGTTTGGCTGGGGTA
PLAT (Hs)	AGGGCTGGAGAGAAAACCTC	CGAAACGAAGACTGCTCCAC
SMAD3 (Hs)	GTGACCACCAGATGAACCAC	GTAGTAGGAGATGGAGCACC
DLK1 (Hs)	GTCCCCTTTGTGACCAGTGC	GAGGAGCAGGCCCGAACATC
*β*2M (Mm)	TGGTGCTTGTCTCACTGACC	CGGGTGGAACTGTGTTACG
RAR*β*2 (Mm)	ACAAGTCATCGGGCTACCAC	CAGTACTGGCATCGGTTCCT
CYP26A1 (Mm)	ACCCACATGTCCTCCAGAAA	AGGATTCAATCGCAGGGTCT
CRBP1 (Mm)	GGACTTCAACGGGTACTGGA	AGTTGGCGATTTTGCGTAAG
DUSP6 (Mm)	AGTTTTTCCCTGAGGCCATT	CATCGTTCATGGACAGGTTG
PLAT (Mm)	ACTCAGTGCCTGTCCGAAGT	GCACTGGCAGACAAAGTCAG
RGS16 (Mm)	GCTCCGATACTGGGGGTATT	CGTCTTTAGGAAGGCATGGA
FLNB (Mm)	CCCAAACTCAACCCAAAGAA	CCTTCTGGGTCCTCAACAAA
EGR1 (Mm)	GAGCGAACAACCCTATGAGC	GGGATAACTCGTCTCCACCA
RET (Mm)	ACAAGAGGCCAGTGTTTGCT	GTGAGTCCGAAGGTGTGGAT

**Table 2:** Comparison of target gene fold induction by aRA between microarray data (outside parenthesis) and competitive RT–PCR results (inside parenthesis) in neuroblastoma cells treated with 10 *μ*M aRA

		**SH-SY5Y**				**BE(2)-C**			
**Gene description**	**GeneBank accession**	**1 h**	**24 h**	**3 days**	**7 days**	**1 h**	**24 h**	**3 days**	**7 days**
RGS16	AA453774	2.0 (2.4)	4.0 (4.1)	3.0 (2.9)	2.2 (1.9)	1.7 (1.9)	2.9 (3.2)	1.9 (2.6)	1.7 (1.9)
TIA1	N59426	0.2 (0.9)	0.1 (0.8)	0.2 (0.9)	0.2 (0.9)	0.6 (1.2)	0.6 (0.7)	0.6 (0.8)	0.5 (0.6)
RAR*β*2	AA419238	0.9	0.9	1.5	2.6	3.1	4.0	4.0	5.4
CYP26A1	R51021	1.1 (—)	11 (91)	11 (100)	17 (94)	0.8 (—)	1.5 (36)	3.3 (87)	7.3 (94)
CRBP1	AA700832	1.2 (1.0)	2.2 (1.9)	3.0 (1.7)	3.2 (1.6)	0.8 (1.2)	2.0 (1.6)	2.9 (2.8)	3.6 (2.3)
CRABP1	AA454702	1.1	2.6	2.4	4.3	1.0	1.2	2.2	5.1
ATP7A	AA236141	1.0 (1.7)	2.0 (2.6)	4.5 (4.2)	7.2 (3.9)	1.0 (1.1)	2.1 (4.6)	3.9 (16)	6.3 (23)
IL13RA2	R52796	1.1	1.2	1.7	5.1	1.0	2.0	2.0	3.2
DUSP6	AA630374	1.2	3.0	5.5	5.5	0.8	3.5	6.7	5.9
EGR1	AA486533	1.0 (1.0)	2.4 (3.4)	2.6 (1.3)	3.4 (1.3)	1.4 (0.3)	2.6 (1.2)	2.0 (1.3)	1.9 (1.5)
RET	H24956	1.2 (0.9)	3.3 (2.7)	4.0 (2.3)	4.0 (1.8)	1.2 (0.7)	2.5 (1.4)	2.9 (2.7)	2.3 (2.1)
PLAT	AA447797	0.9 (1.4)	3.1 (1.5)	3.3 (1.8)	17 (2.0)	1.0 (1.4)	2.0 (1.9)	3.9 (2.3)	6.1 (2.4)
FLNB	AA486239	1.1 (1.2)	2.1 (5.2)	3.2 (4.2)	8.5 (6.0)	1.2 (0.7)	3.7 (2.0)	4.3 (2.5)	10 (3.2)
ITGA1	H68922	1.1	2.4	3.4	6.5	0.9	2.4	3.5	3.7
TMSB4X	AA634103	1.3	2.3	3.7	8.3	1.0	1.4	3.4	4.5
HOXD9	AA424871	1.2	2.0	2.5	3.4	1.1	1.5	1.7	2.0
CNN2	AA284856	1.2	2.2	1.9	3.5	0.9	1.0	1.6	2.1
TRB2	AA458653	1.3	2.2	4.0	6.3	1.3	1.8	2.7	3.4
CXCR4	AA621824	1.1	0.5	0.4	0.4	1.0	1.0	0.5	0.5
TM4SF3	AA045699	1.1	1.3	2.8	3.8	1.2	1.1	2.7	6.0
DCX	AA620421	1.0	1.1	2.9	3.1	1.1	1.3	3.8	3.4
ASL	AA486741	1.1	1.9	3.0	3.2	1.1	1.8	2.9	2.5
DCN	AA099394	1.1	1.3	2.6	2.5	1.1	1.1	1.9	2.8
TP53I3	AA668595	1.1	1.1	1.6	3.0	1.2	1.4	2.9	5.1
IGFBP3	AA598601	1.1	0.5	0.8	3.2	0.9	0.9	2.4	4.6
AF1Q	AA456008	1.3	1.7	2.8	3.4	1.2	1.5	1.9	2.8
DLK1	AA576129	1.5 (0.9)	0.6 (0.7)	0.5 (0.6)	0.2 (0.4)	1.0 (1.1)	1.2 (1.0)	0.3 (0.7)	0.1 (0.5)
SGNE1	AA670429	1.1	1.1	1.5	3.1	0.9	1.0	1.7	2.7
PTN	AA001449	1.2	1.0	1.5	2.9	1.0	1.2	1.8	2.5
SMAD3	W72201	1.1 (1.2)	1.2 (2.7)	1.8 (2.1)	3.1 (2.1)	0.9 (1.1)	1.4 (1.4)	1.6 (1.7)	3.0 (2.7)
PTPRU	AA644448	1.2	0.7	0.6	0.5	1.2	0.7	0.6	0.4

(—) represents undetectable by PCR.

**Table 3:** Target gene fold induction by aRA in lung and breast cancer cells treated with 10 *μ*M aRA

		**Calu6 (SK-MES-1)**				**T47D (MDA-MB-231)**			
**Gene description**	**GeneBank accession**	**1 h**	**24 h**	**3 days**	**7 days**	**1 h**	**24 h**	**3 days**	**7 days**
RGS16	AA453774	2.1 (—)	1.4 (—)	1.6 (—)	1.4 (—)	3.2 (—)	2.7 (—)	1.7 (—)	1.4 (—)
RAR*β*2	AA419238	1.5 (—)	7.5 (—)	8.6 (—)	9.8 (—)	1.2 (—)	12 (—)	13 (—)	22 (—)
CYP26A1	R51021	2.7 (—)	7.0 (—)	7.1 (—)	7.1 (—)	1.1 (—)	11 (—)	11 (—)	17 (—)
CRBP1	AA700832	1.1 (1.5)	1.4 (1.6)	1.5 (1.7)	2.0 (2.0)	1.5 (1.5)	2.5 (1.6)	2.7 (1.9)	5.3 (2.0)
ATP7A	AA236141	1.1 (0.9)	1.1 (0.8)	0.9 (1.0)	1.3 (1.0)	1.1 (1.0)	1.1 (1.1)	0.9 (1.2)	1.3 (1.1)
DUSP6	AA630374	1.1 (1.1)	1.3 (1.1)	1.4 (1.1)	1.4 (1.3)	1.1 (0.9)	1.4 (1.0)	1.6 (1.0)	2.0 (0.9)
EGR1	AA486533	4.0 (—)	0.8 (—)	1.5 (—)	1.5 (—)	— (—)	— (—)	— (—)	— (—)
RET	H24956	1.0 (0.9)	0.6 (0.8)	0.1 (1.0)	0.1 (0.9)	1.5 (—)	1.6 (—)	1.6 (—)	1.6 (—)
PLAT	AA447797	1.0 (0.9)	1.0 (1.0)	0.5 (1.0)	0.5 (0.8)	0.8 (0.9)	1.5 (1.0)	1.6 (0.8)	1.5 (0.9)
FLNB	AA486239	1.0 (1.0)	1.2 (1.2)	1.7 (1.1)	1.7 (1.0)	1.1 (1.1)	1.1 (1.3)	1.3 (1.0)	1.2 (1.0)
DLK1	AA576129	— (—)	— (—)	— (—)	— (—)	1.0 (—)	0.9 (—)	0.7 (—)	0.8 (—)
SMAD3	W72201	1.0 (1.0)	1.0 (1.0)	1.0 (1.0)	1.0 (1.0)	1.1 (1.0)	1.1 (1.0)	1.0 (1.0)	1.0 (0.9)

Calu6 and T47D cells are RA sensitive, while SK-MES-1 and MDA-MB-231 are RA resistant. (—) represents undetectable by PCR.
